# Association of body fat percentage with kidney stone Disease: a cross-sectional and longitudinal study among bus drivers

**DOI:** 10.1186/s12889-023-17128-y

**Published:** 2023-11-06

**Authors:** Xujuan Zheng, Qianqian Chen, Yanxia Wu, Juan Xiong

**Affiliations:** 1 Medical School, Shenzhen University, 1066 Xueyuan Avenue, Nanshan District, Shenzhen, Guangdong Province 518060 China; 2 Health Management Centre, Shenzhen People’s Hospital (The Second Clinical Medical College, Jinan University, The First Affiliated Hospital, Southern University of Science and Technology), Shenzhen, Guangdong Province China

**Keywords:** Kidney stone, Body fat percentage, Obesity, Risk factor, Cross-sectional and longitudinal study

## Abstract

**Background:**

The association between body fat percentage (BFP) and kidney stone disease (KSD) among bus drivers has not been explored in the existing literature. Thus, this study was conducted to explore the influence of BFP on the risk of KSD as well as KSD development for bus drivers to fill the research gap.

**Methods:**

A cross-sectional and longitudinal cohort study was designed. In total, 3433 bus drivers were included in the cross-sectional analyses, and 1864 bus drivers without KSD at baseline and with regular follow-up were included in the longitudinal cohort study.

**Results:**

During a median follow-up of 2.9 years, KSD occurred in 15.0% of bus drivers. Multivariate logistic analysis found that each 5% higher BFP was not only significantly related with higher odds of KSD (odds ratio [OR] = 1.48), but also associated with higher odds of developing KSD (OR = 1.27). The risk of prevalent KSD in obesity group based on BFP was 2.47 times of the normal group; and the corresponding risk of developing KSD was 1.61 times. For obesity bus drives with age < 40, the corresponding risk increased to 4.54 times.

**Conclusion:**

Bus drivers were reported to have a high prevalence of KSD as well as development of KSD. As a significant predictive factor for KSD, BFP can be used to monitor and prevent bus drivers from kidney stone formation. Bus drivers in obesity group based on BFP, especially with age < 40 years should become priority subjects for targeted prevention.

**Supplementary Information:**

The online version contains supplementary material available at 10.1186/s12889-023-17128-y.

## Introduction

Nephrolithiasis, commonly known as kidney stone disease (KSD) or renal calculi, is a condition characterized by the formation of stones within the kidney [1]. The incidence rate of KSD has been steadily increasing worldwide over the past few decades [2]. For instance, in mainland China, the prevalence of KSD has shown an upward trend. Between 1991 and 2000, the prevalence was 6.0%, which increased to 8.9% between 2001 and 2010, and further rose to 10.6% from 2011 to the present [3]. Similarly in the United States, the incidence of KSD has also been on the rise. In 1980, the prevalence was 3.2%, and by 2016, it had increased to 10.1% [4]. Recurrence of KSD is also common, with approximately 50% of patients experiencing a recurrence within ten years, and those with recurrent stones are more likely to suffer from further recurrences [5]. Nephrolithiasis is a global health issue, with an estimated cost of over four billion dollars by 2030 [6]. In addition to the economic burden, KSD can have a significant impact on patient’s quality of life due to common symptoms of pain, anxiety, fatigue, and insomnia [7]. Furthermore, KSD can lead to complications such as chronic and end-stage kidney disease [8].

A number of factors have been suggested as potential causes of kidney stone development, but occupational factors have not received much attention [9]. Certain occupations may make it difficult for individuals to consume enough fluids to maintain diluted urine [9], which is a crucial factor in preventing kidney stone formation [10]. For example, bus drivers usually encounter poor access to fluids or bathroom facilities, which could render them to restrict water uptake, thereby increasing their risk for KSD [11]. Therefore, more attention needs to be paid to the potential link between occupation and KSD, particularly in high-risk occupations such as bus drivers. The national survey in 2020 reported that there were almost one million bus drivers in China, and bus drivers aged 41–50 years accounted for 52.3%, and more than 80.0% was male [12].

Obesity has emerged as a significant global health concern in recent times [13]. Extensive research has provided robust evidence establishing a linkage between obesity and an increased risk of KSD [14–16]. Body mass index (BMI) defined as body weight (kg)/body height (m)^2^, has been widely used as an indicator of obesity in research exploring the association of obesity and KSD due to its low cost and measure simplicity [17]. However, BMI has limitations in accurately distinguishing between adipose and non-adipose tissues [18]. Further, studies have found that BMI is a poor predictor of body fatness due to its low sensitivity [19]. Instead, body fat percentage (BFP), defined as the ratio of total fat mass to total body mass, provides a more precise review of body fat composition [20] that may overcome the limitation of BMI [13]. In the last two decades, the World Health Organization (WHO) has recognized the discrepancy between BMI and BFP, especially in the Asian population where individuals tend to exhibit a higher BFP for the same BMI compared to other ethnicities [21]. Thus, it is reasonable to posit that BFP as a more appropriate indicator of obesity [13], may also be a more precise predictive factor for development of KSD in clinical practice.

A literature survey has led us to find only one study that explored the association between body fat mass and KSD in US adults [22]. One limitation with this particular study, however, is that it relied on self-reporting data for the diagnosis of KSD, which may have recall bias and inaccurate determination of the disease. Furthermore, the cross-sectional design of the study could not establish a causal relationship between BFP and the development of KSD. Moreover, the related existing literature did not involve the special occupation of bus drivers. In this context, we endeavored to address this knowledge gap by designing a cross-sectional and longitudinal study to explore the influence of BFP on the risk of KSD and its development in Chinese bus drivers.

## Materials and methods

### Study design and population

The present study utilizes a cross-sectional and longitudinal cohort design to investigate the influence of BFP on the risk of KSD and its development specifically among bus drivers. We conducted a secondary analysis using existing data sets that were collected during annual health examinations of bus drivers at a hospital in Shenzhen City, China, between 2017 and 2021. The data was analyzed to primarily investigate different blood pressure states transitions among bus drivers, and the details of data collection have been described previously [23].

Inclusion criteria were: (1) bus drivers who have worked for at least two years; (2) subjects with at least two annual health checkup records. Exclusion criteria were: (1) subjects with diagnosed kidney disease or glomerular filtration rate (eGFR) < 60 mL/min/1.73m^2^; (2) subjects with other severe physical or mental diseases, such as carcinoma, serious liver dysfunction, several cardiovascular or cerebral disease, serious depression or anxiety. All study participants signed a consent form before participation. The project followed the Declaration of Helsinki and was approved by the ethics committee of one hospital.

**Fig. 1:**
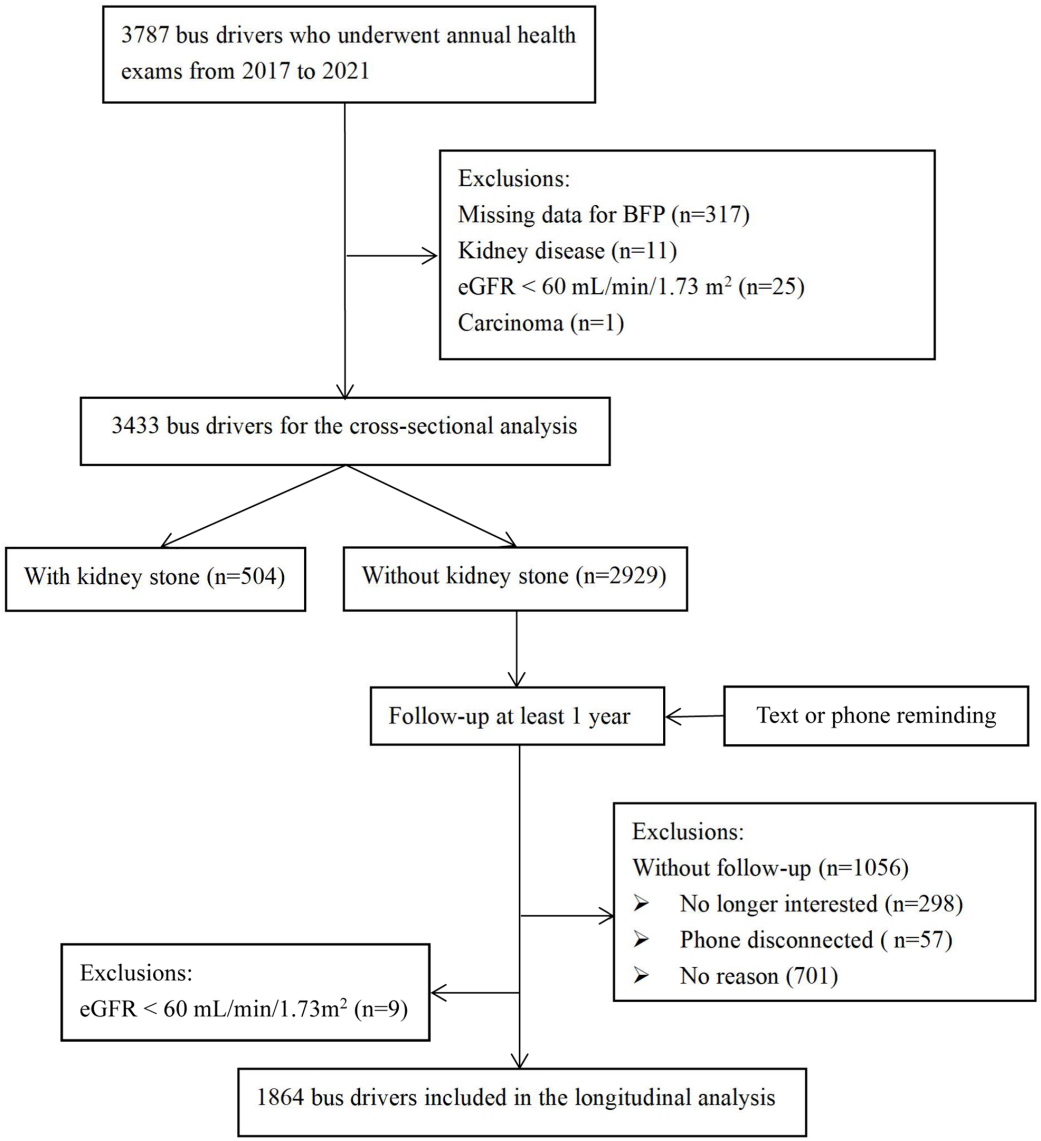
Flow chart of our study population selection

Figure 1 illustrates the flow chart of our study population selection, a total of 3433 subjects were included in the cross-sectional analysis. As one subgroup of the main cohort, 2929 subjects without KSD at baseline were screened for the longitudinal cohort, and 1056 subjects without follow-up visit and 9 subjects with eGFR < 60 mL/min/1.73 m^2^ were excluded. Finally, 1864 subjects with at least one follow-up were enrolled in the longitudinal analysis. The median follow-up time was 2.9 years.

Figure 1: A total of 3787 subjects having an annual health examination from 2017 to 2021 were initially screened for cross-sectional cohort. Of which, 317 individuals with missing data for BFP, 11 individuals with diagnosed kidney disease, 25 individuals with eGFR < 60 mL/min/1.73 m^2^, and one individual diagnosed with carcinoma were excluded.

### KSD and BFP

In the current research, all of the bus drivers with KSD were diagnosed by ultrasound, and likewise were asked to report their feelings to match their stone episodes (i.e., about 90% reported low back pain or discomfort, and 10% reported no obvious symptoms). Ultrasounds of all participants were conducted by professional sonographers in the same hospital to make sure the measurement methods were consistent in the different BFP groups. Meanwhile, these sonographers were blinded to the BFR grouping. Bioelectrical impedance analysis (BIA) (MC-980 Body Composition Analyzer, Tanita, Inc., Shanghai, China) was conducted to measure BFP for bus drivers. BIA had been widely used to estimate body composition [24]. Compared with other measuring equipment, such as dual-energy X-ray absorptiometry (DXA), computerized tomography or magnetic resonance imaging, BIA is more mobile, less costly and less risky in terms of radiation exposure [24]. To keep consistency, the same type of device and brand of BIA was used for measuring BFP, which shows high measurement precision, with its correlation coefficient with DXA exceeding 0.9 according to its instruction manual. For men, individuals with BFP at 10-20%, 21-25%, and > 25% were recommended as normal category, risk for obesity, and obesity; while for women, subjects with BFP at 20-30%, 31-35%, and > 35% were regarded as the corresponding categories [25].

### Covariates

A variety of covariates were likewise collected. During the health checkup, participants’ age and sex were recorded, and their systolic blood pressure (SBP), diastolic blood pressure (DBP), height and weight were measured by professional health workers. The information of comorbidities included whether subjects were diagnosed by dyslipidemia, diabetes, and hypertension. The data of laboratory parameters comprised estimated glomerular filtration rate (eGFR), uric acid, creatinine, blood urea nitrogen (BUN), albumin, total cholesterol, triglyceride, glucose, alanine aminotransferase (ALT), aspartate aminotransferase (AST), and urine pondus hydrogenii (UPH) by blood test and urine test.

### Statistical methods

All statistical analyses were performed via R 4.3.1 software (R Core Team, 2023) [26], the *stats* (V4.3.1) [26] and the *tidyverse* (V2.0.0) packages [27]. *P-*values < 0.05 (two-tailed) were considered statistically significant. Numbers, percentages (%) were used to describe categorical variables; and mean, standard deviation (SD) were used to describe continuous data. *X*^*2*^ test for categorical variables and t-test or ANOVA (Analysis of Variance) for continuous variables were conducted to make comparisons in terms of socio-demographic and clinical characteristics between groups according to the presence of KSD and among different BFP groups. Multivariate logistic regression models were used to assess the association between BFP and the risk of KSD by evaluating the value of odds ratio (OR) and 95% confidence interval (CI). These models included known potential confounders between BFP and nephrolithiasis, as well as covariates in univariate analysis (*P* < 0.20). Three models were constructed: model 1 adjusted for age and sex, model 2 adjusted for sex, age and uric acid, and model 3 adjusted for sex, age, uric acid, eGFR, total cholesterol, triglyceride, diabetes and hypertension.

## Results

### The profile of bus drivers in the cross-sectional cohort

Table 1 presents a comparison of socio-demographic and clinical characteristics between subjects with and without KSD in the cross-sectional cohort. The mean age of the participants was 41.7 (6.91), and a majority (79.1%) of them being male. Overall, 14.7% (504/3433) of bus drivers had KSD diagnosed via ultrasounds, and 17.0% (461/2714) of male and 6.0% (43/719) of female were kidney stone formers. Participants with KSD exhibited several significant differences compared to those without KSD, including higher age, higher systolic and diastolic blood pressure, as well as higher incidence of hypertension, BMI, BFP, uric acid, hemoglobin, creatinine, BUN, total cholesterol, triglyceride, ALT, and UPH. Additionally, participants with KSD had lower eGFR level (*P* < 0.05).

**Table 1:** Socio-demographic and clinical characteristics of bus drivers in the cross-sectional cohort classified by with and without KSD

	All subjects N = 3433	Kidney stone (-) N = 2929	Kidney stone (+) N = 504	*p* value
Age (year)^a^	41.7 (6.9)	41.2 (7.1)	44.6 (5.1)	< 0.001
**Sex group** ^b^				< 0.001
Female	719 (20.9%)	676 (23.1%)	43 (8.5%)	
Male	2714 (79.1%)	2253 (76.9%)	461 (91.5%)	
Systolic BP (mmHg) ^a^	124.7 (16.8)	123.9 (16.4)	129.3 (18.3)	< 0.001
Diastolic BP (mmHg) ^a^	77.0 (11.8)	76.4 (11.6)	80.6 (12.3)	< 0.001
**Comorbidities**				
Dyslipidemia ^b^	1309 (71.5%)	1069 (71.1%)	240 (73.2%)	0.499
Diabetes ^b^	176 (5.1%)	145 (5.0%)	31 (6.2%)	0.308
Hypertension ^b^	875 (25.5%)	722 (24.7%)	153 (30.4%)	0.007
**Obesity-related index**				
BFP (%)^a^	24.5 (5.4)	24.4 (5.6)	24.9 (4.4)	0.018
BFP group ^b^				< 0.001
Normal category	966 (28.1%)	898 (30.7%)	68 (13.5%)	
Risk for obesity	1442 (42.0%)	1216 (41.5%)	226 (44.8%)	
Obesity	1025 (29.9%)	815 (27.8%)	210 (41.7%)	
**Laboratory parameters**				
eGFR (mL/min/1.73m^2^) ^a^	102.8 (18.8)	103.6 (17.9)	98.3 (23.1)	< 0.001
Uric acid (mg/dL) ^a^	6.4 (1.6)	6.4 (1.6)	6.9 (1.6)	< 0.001
Hemoglobin (g/L) ^a^	153.5 (16.1)	153.1 (16.4)	155.8 (13.4)	< 0.001
Creatinine (µmoI/L) ^a^	0.9 (0.2)	0.8 (0.2)	0.9 (0.2)	< 0.001
BUN (mg/dL) ^a^	12.0 (3.0)	11.9 (3.0)	12.4 (3.0)	0.001
Albumin (g/L) ^a^	46.0 (2.6)	46.0 (2.6)	45.9 (2.4)	0.274
Glucose (mol/L) ^a^	5.4 (1.4)	5.4 (1.4)	5.5 (1.5)	0.088
Total cholesterol (mg/dL) ^a^	199.8 (38.4)	198.8 (38.4)	205.4 (37.8)	< 0.001
Triglyceride (mg/dL) ^a^	171.7 (138.6)	168.2 (134.8)	192.1 (157.4)	0.001
ALT (U/L) ^a^	29.8 (21.6)	29.3 (21.9)	32.8 (19.5)	< 0.001
AST (U/L) ^a^	24.8 (11.8)	24.7 (12.3)	25.3 (7.9)	0.169
UPH ^b^				0.071
6 ≤ UPH ≤ 7	1681 (49.0%)	1438 (49.1%)	243 (48.2%)	
UPH < 6	1407 (41.0%)	1184 (40.4%)	223 (44.2%)	
UPH > 7	345 (10.0%)	307 (10.5%)	38 (7.5%)	

kidney stone disease (KSD), body fat percentage (BFP), estimated glomerular filtration rate (eGFR), blood urea nitrogen (BUN), alanine aminotransferase (ALT), aspartate aminotransferase (AST), and urine pondus hydrogenii (UPH).

^a^ Data are given as mean (sd), and *P* values were calculated by the t-test

^b^ Data are expressed as number (percentage), and *P* values were calculated by the chi-squared test

**Fig. 2:**
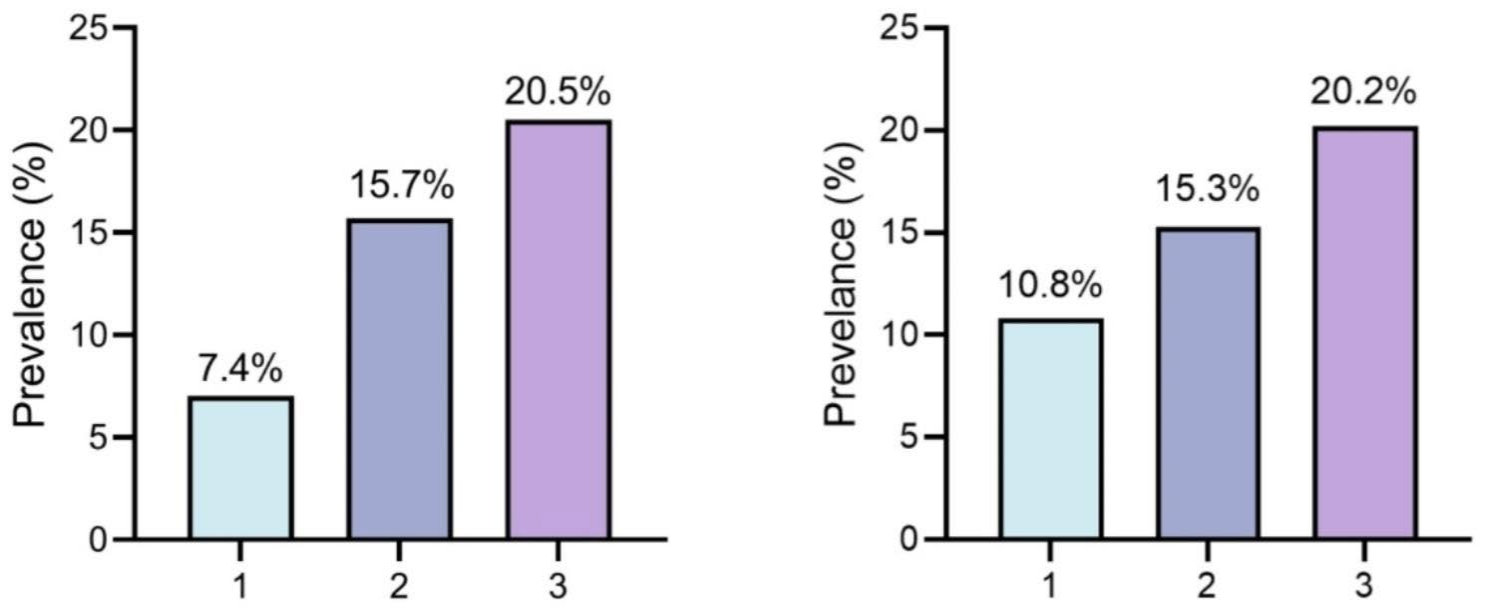
**a**: The prevalence of KSD in different BFP groups; **b** The prevalence of developing KSD in different BFP groups KSD: kidney stone disease; BFP: body fat percentage 1: Normal category, 2: Risk for obesity, 3: Obesity

We further stratified these bus drivers by different BFP group and gender group, and their discrepancies in socio-demographic and clinical characters were described in Table S1 and S2, respectively. As shown in Table S1, 1025 subjects (29.9%) were in obesity group based on BFP, comprising 142 females with BFP > 35% and 883 males with BFP > 25%. The prevalence of KSD in different BFP groups was illustrated in Fig. 2.

Figure 2: The prevalence of KSD in different BFP group, and the prevalence of developing KSD in different BFP group. The BFP groups were 1 normal group, 2 risk for obesity group and 3 obesity group.

Table 2 presents the results of multivariate logistic analysis conducted to explore the association between BFP and prevalent KSD among bus drivers. Our analysis results for model 3 revealed that each 5% increase in BFP was significantly related with higher odds of KSD (OR = 1.48, 95% CI: 1.30–1.67) for all bus drivers, with a more significant effect observed among bus drivers aged < 40 (OR = 1.84, 95% CI: 1.37–2.48). In terms of different BFP groups, the risk of prevalent KSD was 2.47 times higher among bus drivers in the obesity group compared to those in the normal group (OR = 2.47, 95% CI: 1.82–3.37). Notably, bus drives aged < 40 in obesity group had a significantly increased risk of prevalent KSD, with odds that were 4.54 times higher than those in the normal group (OR = 4.54, 95% CI: 2.13–10.60).

**Table 2:** Association of BFP parameter with prevalent KSD among bus drivers stratified by age group (N = 3433)

Odds ratio (95% CI)			
	Model 1	Model 2	Model 3
**All subjects** (N = 3433)			
BFP (per 5%)	1.53 [1.36–1.72]	1.47 [1.30–1.66]	1.48 [1.30–1.67]
BFP group			
Normal	Ref.	Ref.	Ref.
Risk for obesity	2.03 [1.52–2.72]	1.94 [1.46–2.68]	1.94 [1.45–2.61]
Obesity	2.69 [2.01–3.63]	2.45 [1.82–3.34]	2.47 [1.82–3.37]
**Subjects with age < 40 years** (N = 1226)			
BFP (per 5%)	1.80 [1.37–2.36]	1.85 [1.39–2.46]	1.84 [1.37–2.48]
BFP group			
Normal	Ref.	Ref.	Ref.
Risk for obesity	3.78 [1.88–8.44]	3.81 [1.89–8.52]	3.75 [1.86–8.43]
Obesity	4.54 [2.21–10.30]	4.65 [2.22–10.70]	4.54 [2.13–10.60]
**Subjects with age ≥ 40 years** (N = 2207)			
BFP (per 5%)	1.43 [1.25–1.64]	1.36 [1.18–1.56]	1.37 [1.19–1.58]
BFP group			
Normal	Ref.	Ref.	Ref.
Risk for obesity	1.66 [1.21–2.30]	1.57 [1.14–2.18]	1.58 [1.15–2.20]
Obesity	2.25 [1.64–3.13]	2.00 [1.44–2.80]	2.04 [1.46–2.86]

Confidence interval (CI), kidney stone disease (KSD), body fat percentage (BFP), estimated glomerular filtration rate (eGFR)

Model 1: adjusted for age and sex; model 2: adjusted for sex, age and uric acid; and model 3: adjusted for sex, age, uric acid, eGFR, total cholesterol, triglyceride, diabetes and hypertension

### The profile of bus drivers in the longitudinal cohort

To further investigate the association between KSD and BFP, a longitudinal subset of 1864 bus drivers was analyzed. The subjects had a mean age of 41.6, with 1561 (83.7%) being male. Over a median follow-up time of 2.9 years, 279 (15.0%) bus drivers were diagnosed with KSD via ultrasound, including 256 (16.4%) males and 23 (7.6%) females. Table 3 presents a comparison of socio-demographic and clinical characteristics between participants with and without KSD. Those who developed KSD demonstrated several significant differences compared to those who did not, including higher age, higher systolic and diastolic blood pressure, higher incidence of dyslipidemia, BMI, uric acid, hemoglobin, creatinine, BUN, triglyceride, ALT, and lower eGFR level (*P* < 0.05).

**Table 3:** Socio-demographic and clinical characteristics of bus drivers in the longitudinal cohort classified by with and without KSD

	All subjects N = 1864	Kidney stone (-) N = 1585	Kidney stone (+) N = 279	*p* value
Age (year)^a^	41.6 (6.7)	41.4 (6.9)	43.0 (5.3)	< 0.001
**Sex group** ^b^				< 0.001
Female	303 (16.3%)	280 (17.7%)	23 (8.24%)	
Male	1561 (83.7%)	1305 (82.3%)	256 (91.8%)	
Systolic BP (mmHg) ^a^	124.3 (16.2)	124.0 (16.1)	126.4 (16.1)	0.022
Diastolic BP (mmHg) ^a^	76.7 (11.4)	76.3 (11.4)	79.2 (11.2)	< 0.001
**Comorbidities**				
Dyslipidemia ^b^	707 (71.3%)	576 (69.6%)	131 (79.9%)	0.011
Diabetes ^b^	90 (4.8%)	70 (4.4%)	20 (7.2%)	0.068
Hypertension ^b^	454 (24.4%)	381 (24.1%)	73 (26.3%)	0.475
**Obesity-related index**				
BFP ^a^	23.3 (5.0)	23.2 (5.1)	23.8 (4.6)	0.059
BFP group ^b^				< 0.001
Normal	649 (34.8%)	579 (36.5%)	70 (25.1%)	
Risk for obesity	734 (39.4%)	622 (39.2%)	112 (40.1%)	
Obesity	481 (25.8%)	384 (24.2%)	97 (34.8%)	
**Laboratory parameters**				
eGFR (mL/min/1.73m^2^) ^a^	102.7 (16.4)	103.3 (16.4)	99.7 (16.5)	0.001
Uric acid (mg/dL) ^a^	6.5 (1.54)	6.4 (1.5)	6.8 (1.6)	< 0.001
Hemoglobin (g/L) ^a^	155.1 (15.0)	154.7 (15.4)	157.6 (12.6)	0.001
Creatinine (µmoI/L) ^a^	37.4 (31.8)	36.7 (30.8)	41.7 (36.7)	< 0.001
BUN (mg/dL) ^a^	3.3 (0.7)	3.3 (0.7)	3.4(0.7)	0.023
Albumin (g/L) ^a^	2.0 (1.5)	1.9 (1.5)	2.1 (1.4)	0.919
Glucose (mol/L) ^a^	1.2 (0.3)	1.2 (0.3)	1.2 (0.2)	0.315
Total cholesterol (mg/dL) ^a^	199.7 (37.9)	199.3 (38.1)	202.2 (36.8)	0.216
Triglyceride (mg/dL) ^a^	173.3 (135.2)	170.3 (136.7)	189.1 (125.2)	0.023
ALT (U/L) ^a^	30.3 (21.8)	29.9 (21.9)	32.7 (20.7)	0.044
AST (U/L) ^a^	25.2 (11.0)	25.0 (11.2)	26.1 (10.0)	0.094
UPH ^b^				0.155
6 ≤ UPH ≤ 7	25.2 (11.0)	25.0 (11.2)	26.1 (10.0)	
UPH < 6	75.8 (13.0)	75.2 (13.0)	78.7 (12.4)	
UPH > 7	46.2 (2.6)	46.2 (2.6)	46.2 (2.6)	

kidney stone disease (KSD), body fat percentage (BFP), estimated glomerular filtration rate (eGFR), blood urea nitrogen (BUN), alanine aminotransferase (ALT), aspartate aminotransferase (AST), and urine pondus hydrogenii (UPH).

^a^ Data are given as mean (sd), and *P* values were calculated by the t-test

^b^ Data are expressed as number (percentage), and *P* values were calculated by the chi-squared test

The results of stratification analysis by different BFP group are provided in Table S3. Among the 1864 participants, 481(25.8%) were in the obesity group, including 33 females with BFP > 35% and 448 males with BFP > 25%. Of these, 20.2% of individuals in the obesity group developed KSD.

Table 4 presents the findings of multivariate logistic analysis conducted in the longitudinal cohort to explore the association between BFP and the development of KSD. Our analysis results for model 3 revealed that for every 5% increase in BFP, all bus drivers had a 1.27 times higher risk of developing KSD (OR = 1.27, 95% CI: 1.08–1.48). Furthermore, the risk of developing KSD among bus drivers in the obesity group was 1.61 times higher than that among those in the normal group (OR = 1.61, 95% CI: 1.13–2.32). These results suggest that higher BFP is a significant risk factor for developing KSD among bus drivers.

**Table 4:** Association of BFP parameter with developing KSD among bus drivers stratified by age group (N = 1864)

Odds ratio (95% CI)			
	Model 1	Model 2	Model 3
**All subjects** (N = 1864)			
BFP (per 5%)	1.32 [1.14–1.53]	1.27 [1.09–1.48]	1.27 [1.08–1.48]
BFP group			
Normal	Ref.	Ref.	Ref.
Risk for obesity	1.30 [0.94–1.81]	1.25 [0.90–1.74]	1.22 [0.87–1.71]
Obesity	1.80 [1.28–2.54]	1.64 [1.15–2.35]	1.61 [1.13–2.32]
**Subjects with age < 40 years** (N = 664)			
BFP (per 5%)	1.26 [0.96–1.65]	1.25 [0.94–1.67]	1.29 [0.95–1.74]
BFP group			
Normal	Ref.	Ref.	Ref.
Risk for obesity	0.99 [0.53–1.83]	0.99 [0.53–1.85]	1.00 [0.53–1.89]
Obesity	1.85 [0.98–3.48]	1.86 [0.95–3.64]	2.00 [1.00-4.02]
**Subjects with age ≥ 40 years** (N = 1200)			
BFP (per 5%)	1.29 [1.08–1.54]	1.23 [1.02–1.48]	1.21 [1.00-1.47]
BFP group			
Normal	Ref.	Ref.	Ref.
Risk for obesity	1.34 [0.91-2.00]	1.25 [0.84–1.87]	1.22 [0.82–1.84]
Obesity	1.62 [1.08–2.47]	1.44 [0.94–2.22]	1.40 [0.91–2.17]

Confidence interval (CI), kidney stone disease (KSD), body fat percentage (BFP), estimated glomerular filtration rate (eGFR)

Model 1: adjusted for age and sex; model 2: adjusted for sex, age and uric acid; and model 3: adjusted for sex, age, uric acid, eGFR, total cholesterol, triglyceride, diabetes and hypertension

## Discussion

The association between BFP and KSD among bus drivers has not been explored in the existing literature. To fill this research gap, we designed a cross-sectional and longitudinal study to explore the influence of BFP on the risk of KSD and its development for bus drivers. Several significant findings have emerged from our study. First, we observed a higher prevalence of kidney stones among bus drivers, with approximately 14.7% of participants being diagnosed with KSD between 2017 and 2020. In this context, we note that Lee et al. reported that about 6.4% of Taiwan adults had KSD by self-reported from 2008 to 2020 [17], while another study found that 7.8% of American adults self-reported having KSD [22]. Furthermore, our study revealed that after almost three years’ follow-up, around 15% of bus drivers developed KSD, much higher than the previous research findings that described during a mean follow-up of about four years, in which it was shown that only 2.5% of Taiwan adults developed KSD [17]. Our study further highlighted the increased susceptibility of bus drivers to develop KSD compared to other occupational groups. This emphasizes the need for heightened attention and concern from researchers, governments and bus companies regarding the health risks faced by bus drivers.

The high prevalence of KSD among bus drivers can be attributed to several factors, which are discussed below. Firstly, bus drivers often face limited access to fluids or bathroom facilities, leading to inadequate fluid intake and reduced urine volume [9]. Insufficient fluid intake is considered a crucial risk factor for KSD, as it hampers the dilution of urine [11]. Consequently, these bus drivers are more prone to developing KSD due to low urine volume. Secondly, bus drivers in Shenzhen City, where the study was conducted, work in a subtropical climate characterized by long, hot, and humid summers. The ambient temperature, whether related to workplace, or climate, can have a significant effect on stone formation [9]. Factors such as increased sweating and dehydration in hot weather conditions can further contribute to the development of kidney stones among bus drivers. Thirdly, our study revealed that bus drivers had a higher prevalence of dyslipidemia, hypertension, and diabetes. Previous studies has established that these conditions are all independently associated with stone disease [9].

Our study also found that higher BFP was significantly associated with a higher prevalence of KSD and the development of KSD among bus drivers, even after adjusting for covariates. Specifically, we found that for every 5% increase in BFP, there was a significant increase in the odds of having KSD (OR = 1.48, 95% CI: 1.30–1.67) and developing KSD (OR = 1.27, 95% CI: 1.08–1.48). A previous cross-sectional study also found that higher fat percentage was associated with higher odds of KSD among US adults, but did not explore the relationship between BFP and the development of KSD [22]. To the best of our knowledge, our study is the first to investigate the influence of BFP on the development of KSD. Our findings suggest that BFP is a significant predictive factor for KSD development and can be used to monitor and prevent kidney stone formation among bus drivers in clinical practice.

In terms of different age groups, our research found that BFP was associated with a higher prevalence of KSD in bus drivers aged < 40 years. While the other study reported that total and truncal body fay were related with a higher incidence of KSD in US adults who are ≥ 40 years [22]. Some factors such as various body fat parameters and measurements, and different working population could account for the inconsistent findings between the two studies. Further research is needed to verify the present study findings. With regard to different BFP groups, our study revealed that bus drivers in obesity group had the highest prevalence and development of KSD in comparison with other groups. For example, about one fifth of the bus drivers in obesity group had KSD or developed KSD; while only approximately one tenth in normal group had KSD or developed KSD. Therefore, as the subjects with high incidence of KSD, bus drivers in obesity group, especially those aged < 40 should become priority groups for targeted prevention and investigation.

The pathophysiology underlying the association between higher BFP and KSD remains unclear [22], but several factors may contribute to it. Firstly, obesity could lead to urinary tract infections, which are well-recognized risk factor for kidney stone formation [28]. Secondly, obesity can alter urine composition, and obesity subjects with KSD are reported to have hypercalciuria, hyperoxaluria, and hyperphosphate, which are indeed important risk factors for kidney stone [29, 30]. Thirdly, obesity could cause lower urinary PH, which is a major factor in the development of KSD [31]. Fourthly, obesity is related to insulin resistance, which can lead to lower citrate levels in urine, a significant inhibitor of kidney stone formation [32].

In summary, the current research findings indicated that bus drivers as a higher risk occupation group for KSD should deserve more attention and concern from the Chinese governments, bus companies and researchers. In particular, these obesity bus driver defined based on BFP with age < 40 years deserve priority attention for targeted prevention and investigation. In addition, BFP as one precise predictive factor for KSD could be used to monitor and prevent bus drivers from kidney stone formation in clinical practice.

Our study has several notable strengths that contribute to its significance, which include a cross-sectional and longitudinal study design, unique exploration of the influence of BFP on the risk and development of KSD, and a focus on the special occupational group of bus drivers. Notwithstanding, some limitations of this study is also worthy of mention. One limitation is the gender disparity among the participants, with the majority being male bus drivers. This gender imbalance reflects the existing gender ratio gap within this occupation [12]. The small proportion of female bus drivers in our study made it impossible to conduct stratification analysis by gender in the present study. Furthermore, owing to the nature of longitudinal data, we experienced a significant loss of participants (36.1%) during the follow-up period, probably leading to attrition bias. Moreover, information bias could be caused from the diagnosis of KSD by ultrasound that may overestimate the presence of urinary calculi, and the BFP measurement using BIA that may result in measurement inaccuracies. In addition, some covariates relating to the lifestyle of bus drivers, such as daily fluid intake, smoking status, and exercise were not included in the study, which could affect kidney stone formation and should be explored in further research.

## Conclusion

In the current research, bus drivers were reported to have a high prevalence of KSD as well as development of KSD. As a significant predictive factor for KSD, BFP can be used to monitor and prevent bus drivers from kidney stone formation. Bus drivers in obesity group based on BFP, especially with age < 40 years should become priority subjects for targeted prevention.

### Electronic supplementary material

Below is the link to the electronic supplementary material.

Supplementary Material 1

## Data Availability

The datasets generated during and/or analyzed during the current study are available from the corresponding author on reasonable request.

## Declarations

• Consent for publication.

### Registry and the Registration No. of the study/trial

N/A.

### Animal Studies

N/A.
