# Supplementary material for: Association of body fat percentage with kidney stone Disease: a cross-sectional and longitudinal study among bus drivers
Source: BMC Public Health. 2023 Nov 6;23:2174. doi: 10.1186/s12889-023-17128-y (PMC10629027; doi:10.1186/s12889-023-17128-y)
Supplement: Supplementary file 1 — Supplementary Material 1 [file 12889_2023_17128_MOESM1_ESM.docx]

Table S1 Socio-demographic and clinical characteristics of bus drivers in the cross-sectional cohort classified by BFP group

|  | All subjects  N=3433 | Normal category  N=966 | Risk for obesity based on BFP  N=1442 | Obesity based on BFP  N=1025 | *p* value |
| --- | --- | --- | --- | --- | --- |
| Age (year) ^a^ | 41.7 (6.9) | 39.9 (7.4) | 42.1 (6.7) | 42.8 (6.4) | < .001 |
| **Sex group** ^b^ |  |  |  |  | < .001 |
| Female | 719 (20.9%) | 322 (33.3%) | 255 (17.7%) | 142 (13.9%) |  |
| Male | 2714 (79.1%) | 644 (66.7%) | 1187 (82.3%) | 883 (86.1%) |  |
| Systolic BP (mmHg) ^a^ | 124.7 (16.8) | 118.9 (14.4) | 124.3 (16.8) | 130.7 (17.0) | < .001 |
| Diastolic BP (mmHg) ^a^ | 77.0 (11.8) | 72.7 (10.3) | 76.9 (11.8) | 81.3 (11.7) | < .001 |
| ***Comorbidities*** |  |  |  |  |  |
| Dyslipidemia ^b^ | 1309 (71.5%) | 197 (53.2%) | 595 (74.9%) | 517 (77.5%) | < .001 |
| Diabetes ^b^ | 176 (5.1%) | 24 (2.5%) | 77 (5.3%) | 75 (7.3%) | < .001 |
| Hypertension ^b^ | 875 (25.5%) | 179 (18.5%) | 359 (24.9%) | 337 (32.9%) | < .001 |
| KSD ^b^ | 504 (14.7%) | 68 (7.0%) | 226 (15.7%) | 210 (20.5%) | < .001 |
| ***Laboratory parameters*** |  |  |  |  |  |
| eGFR (mL/min/1.73m^2^) ^a^ | 102.8 (18.8) | 106.6 (18.0) | 102.0 (17.1) | 100.3 (21.2) | < .001 |
| Uric acid (mg/dL) ^a^ | 6.4 (1.6) | 5.8 (1.4) | 6.4 (1.5) | 7.0 (1.6) | < .001 |
| Hemoglobin (g/L) ^a^ | 153.5 (16.1) | 149.2 (17.0) | 154.0 (15.6) | 156.8 (14.9) | < .001 |
| Creatinine (μmoI/L) ^a^ | 0.9 (0.2) | 0.8 (0.2) | 0.9 (0.2) | 0.9 (0.2) | < .001 |
| BUN (mg/dL) ^a^ | 12.0 (3.0) | 11.7 (3.0) | 12.0 (3.0) | 12.1 (2.9) | .007 |
| Albumin (g/L) ^a^ | 46.0 (2.6) | 46.1 (2.7) | 46.1 (2.5) | 45.8 (2.6) | .018 |
| Glucose (mg/dL) ^a^ | 5.4 (1.4) | 5.3 (1.4) | 5.4 (1.4) | 5.6 (1.4) | < .001 |
| Total cholesterol (mg/dL) ^a^ | 199.8 (38.4) | 192.6 (37.5) | 201.1 (38.8) | 204.8 (37.6) | < .001 |
| Triglyceride (mg/dL) ^a^ | 171.7 (139) | 127.3 (86.8) | 177.1 (147) | 206.0 (154.0) | < .001 |
| ALT (U/L) ^a^ | 29.8 (21.6) | 22.4 (16.0) | 29.3 (19.9) | 37.6 (25.7) | < .001 |
| AST (U/L) ^a^ | 24.8 (11.8) | 22.7 (9.14) | 24.5 (12.7) | 27.1 (12.2) | < .001 |
| UPH ^b^ |  |  |  |  | < .001 |
| 6 ≤ UPH ≤ 7 | 1681 (49.0%) | 484 (50.1%) | 726 (50.3%) | 471 (46.0%) |  |
| UPH < 6 | 1407 (41.0%) | 356 (36.9%) | 566 (39.3%) | 485 (47.3%) |  |
| UPH > 7 | 345 (10.0%) | 126 (13.0%) | 150 (10.4%) | 69 (6.7%) |  |

kidney stone disease (KSD), body fat percentage (BFP), estimated glomerular filtration rate (eGFR), blood urea nitrogen (BUN), alanine aminotransferase (ALT), aspartate aminotransferase (AST), and urine pondus hydrogenii (UPH).

^a^ Data are given as mean (sd), and *P* values were calculated by the ANOVA
^b^ Data are expressed as number (percentage), and *P* values were calculated by the chi-squared test

Table S2 Socio-demographic and clinical characteristics of bus drivers in the cross-sectional cohort classified by gender

|  | All subjects N=3433 | Female N=719 | Male N=2714 | *p* value |
| --- | --- | --- | --- | --- |
| Age (year) ^a^ | 41.7 (6.9) | 37.6 (5.9) | 42.8 (6.7) | < .001 |
| Systolic BP (mmHg) ^a^ | 124.7 (16.8) | 115.7 (14.5) | 127.1 (16.6) | < .001 |
| Diastolic BP (mmHg) ^a^ | 77.0 (11.8) | 70.2 (9.9) | 78.8 (11.6) | < .001 |
| ***Comorbidities*** |  |  |  |  |
| Dyslipidemia ^b^ | 1309 (71.5%) | 103 (64.4%) | 1206 (72.2%) | .046 |
| Diabetes ^b^ | 176 (5.1%) | 18 (2.5%) | 158 (5.8%) | < .001 |
| Hypertension ^b^ | 875 (25.5%) | 142 (19.7%) | 733 (27.0%) | < .001 |
| KSD ^b^ | 504 (14.7%) | 43 (6.0%) | 461 (17.0%) |  |
| ***Obesity-related index*** |  |  |  |  |
| BFP (%)^a^ | 24.5 (5.4) | 30.4 (5.5) | 22.9 (4.2) | < .001 |
| BFP group ^b^ |  |  |  | < .001 |
| Normal | 966 (28.1%) | 322 (44.8%) | 644 (23.7%) |  |
| Risk for obesity | 1442 (42.0%) | 255 (35.5%) | 1187 (43.7%) |  |
| Obesity | 1025 (29.9%) | 142 (19.7%) | 883 (32.5%) |  |
| BMI (kg/m^2^) ^a^ | 25.0 (3.4) | 23.6 (3.1) | 25.3 (3.4) | < .001 |
| BMI group ^b^ |  |  |  | < .001 |
| <24 kg/m^2^ | 1358 (39.6%) | 435 (60.5%) | 923 (34.0%) |  |
| 24 - 28 kg/m^2^ | 1496 (43.6%) | 222 (30.9%) | 1274 (46.9%) |  |
| >28 kg/m^2^ | 579 (16.9%) | 62 (8.62%) | 517 (19.0%) |  |
| ***Laboratory parameters*** |  |  |  |  |
| eGFR (mL/min/1.73m^2^) ^a^ | 102.8 (18.8) | 114.5 (18.0) | 99.7 (17.8) | < .001 |
| Uric acid (mg/dL) ^a^ | 6.4 (1.6) | 5.0 (1.13) | 6.8 (1.5) | < .001 |
| Hemoglobin (g/L) ^a^ | 153.5 (16.1) | 131.8 (13.4) | 159.2 (11.0) | < .001 |
| Creatinine (μmoI/L) ^a^ | 0.9 (0.2) | 0.63 (0.1) | 0.9 (0.1) | .001 |
| BUN (mg/dL) ^a^ | 12.0 (3.0) | 10.5 (2.8) | 12.3 (2.9) | < .001 |
| Albumin (g/L) ^a^ | 46.0 (2.6) | 44.6 (2.5) | 46.4 (2.5) | < .001 |
| Glucose (mol/L) ^a^ | 5.4 (1.4) | 5.2 (1.0) | 5.5 (1.5) | < .001 |
| Total cholesterol (mg/dL) ^a^ | 119.8 (38.4) | 186.2 (33.8) | 203.4 (38.7) | < .001 |
| Triglyceride (mg/dL) ^a^ | 171.7 (138.6) | 112.3 (73.2) | 187.5 (147.3) | < .001 |
| ALT (U/L) ^a^ | 29.8 (21.6) | 18.0 (12.4) | 32.9 (22.5) | < .001 |
| AST (U/L) ^a^ | 24.8 (11.8) | 20.6 (8.11) | 25.9 (12.3) | < .001 |
| UPH ^b^ |  |  |  | < .001 |
| 6 ≤ UPH ≤ 7 | 1681 (49.0%) | 374 (52.0%) | 1307 (48.2%) |  |
| UPH < 6 | 1407 (41.0%) | 205 (28.5%) | 1202 (44.3%) |  |
| UPH > 7 | 345 (10.0%) | 140 (19.5%) | 205 (7.6%) |  |

kidney stone disease (KSD), body fat percentage (BFP), estimated glomerular filtration rate (eGFR), blood urea nitrogen (BUN), alanine aminotransferase (ALT), aspartate aminotransferase (AST), and urine pondus hydrogenii (UPH).

^a^ Data are given as mean (sd), and *P* values were calculated by the t-test
^b^ Data are expressed as number (percentage), and *P* values were calculated by the chi-squared test

Table S3 Socio-demographic and clinical characteristics of bus drivers in the longitudinal cohort classified by BFP group

|  | All subjects  N=1864 | Normal category  N=649 | Risk for obesity based on BFP  N=734 | Obesity based on BFP  N=481 | *p* value |
| --- | --- | --- | --- | --- | --- |
| Age (year) ^a^ | 41.6 (6.7) | 40.7 (7.1) | 42.0 (6.6) | 42.3 (6.1) | < .001 |
| **Sex group** ^b^ |  |  |  |  | < .001 |
| Female | 303 (16.3%) | 192 (29.6%) | 78 (10.6%) | 33 (6.9%) |  |
| Male | 1561 (83.7%) | 457 (70.4%) | 656 (89.4%) | 448 (93.1%) |  |
| Systolic BP (mmHg) ^a^ | 124.3 (16.2) | 119.8 (14.5) | 124.4 (16.1) | 130.4 (16.4) | < .001 |
| Diastolic BP (mmHg) ^a^ | 76.7 (11.4) | 73.2 (10.5) | 76.9 (11.3) | 81.1 (11.0) | < .001 |
| ***Comorbidities*** |  |  |  |  |  |
| Dyslipidemia ^b^ | 707 (71.3%) | 136 (49.5%) | 323 (78.6%) | 248 (81.3%) | < .001 |
| Diabetes ^b^ | 90 (4.8%) | 16 (2.5%) | 39 (5.3%) | 35 (7.3%) | .001 |
| Hypertension ^b^ | 454 (24.4%) | 126 (19.4%) | 178 (24.3%) | 150 (31.2%) | < .001 |
| KSD ^b^ | 279 (15.0%) | 70 (10.8%) | 112 (15.3%) | 97 (20.2%) | < .001 |
| ***Laboratory parameters*** |  |  |  |  |  |
| eGFR (mL/min/1.73m^2^) ^a^ | 102.7 (16.4) | 105.8 (16.7) | 101.4 (16.2) | 100.7 (15.8) | < .001 |
| Uric acid (mg/dL) ^a^ | 6.5 (1.5) | 5.9 (1.4) | 6.59 (1.4) | 7.19 (1.6) | < .001 |
| Hemoglobin (g/L) ^a^ | 155.1 (15.0) | 150.3 (16.8) | 156.7 (14.0) | 159.2 (11.9) | < .001 |
| Creatinine (μmoI/L) ^a^ | 0.9 (0.2) | 0.81(0.6) | 0.9 (0.1) | 0.9 (0.1) | < .001 |
| BUN (mg/dL) ^a^ | 12.0 (3.0) | 11.7 (3.0) | 12.2 (3.0) | 12.1 (2.8) | .017 |
| Albumin (g/L) ^a^ | 46.2 (2.6) | 46.1 (2.7) | 46.3 (2.5) | 46.1 (2.5) | .171 |
| Glucose (mol/L) ^a^ | 5.4(1.5) | 5.3 (1.4) | 5.5 (1.6) | 5.6 (1.4) | .001 |
| Total cholesterol (mg/dL) ^a^ | 199.7 (37.9) | 193.6 (37.7) | 201.8 (37.8) | 204.7 (37.3) | < .001 |
| Triglyceride (mg/dL) ^a^ | 173.1 (135.2) | 129.3 (87.3) | 186.0 (147.8) | 212.7 (151.2) | < .001 |
| ALT (U/L) ^a^ | 30.3 (21.8) | 22.7 (16.2) | 31.1 (19.8) | 39.5 (26.9) | < .001 |
| AST (U/L) ^a^ | 25.2 (11.0) | 22.9 (9.6) | 25.3 (9.1) | 28.0 (14.2) | < .001 |
| UPH ^b^ |  |  |  |  | < .001 |
| 6 ≤ UPH ≤ 7 | 905 (48.6%) | 326 (50.2%) | 360 (49.0%) | 219 (45.5%) |  |
| UPH < 6 | 786 (42.2%) | 239 (36.8%) | 309 (42.1%) | 238 (49.5%) |  |
| UPH > 7 | 173 (9.3%) | 84 (12.9%) | 65 (8.9%) | 24 (5.0%) |  |
| BFP (%)^a^ | 23.3 (5.0) | 19.4 (4.2) | 23.7 (3.2) | 28.1 (3.7) | < .001 |

kidney stone disease (KSD), body fat percentage (BFP), estimated glomerular filtration rate (eGFR), blood urea nitrogen (BUN), alanine aminotransferase (ALT), aspartate aminotransferase (AST), and urine pondus hydrogenii (UPH).

^a^ Data are given as mean (sd), and *P* values were calculated by the ANOVA
^b^ Data are expressed as number (percentage), and *P* values were calculated by the chi-squared test
